# Stigma and intersectionality: a systematic review of systematic reviews across HIV/AIDS, mental illness, and physical disability

**DOI:** 10.1186/s12889-018-5861-3

**Published:** 2018-07-27

**Authors:** Fatimah Jackson-Best, Nancy Edwards

**Affiliations:** 1 I Am One, 58C Cypress Ridge, Union Hall, San Fernando, Trinidad and Tobago; 2 School of Nursing, Faculty of Health Sciences, University of Ottawa, 1 Stewart Street, Room 205, Ottawa, ON K1N 7M9 Canada

**Keywords:** Stigma, Intersectionality, HIV/AIDS, Mental illness, Physical disability

## Abstract

**Background:**

Stigma across HIV/AIDS, mental illness, and physical disability can be co-occurring and may interact with other forms of stigma related to social identities like race, gender, and sexuality. Stigma is especially problematic for people living with these conditions because it can create barriers to accessing necessary social and structural supports, which can intensify their experiences with stigma. This review aims to contribute to the knowledge on stigma by advancing a cross-analysis of HIV/AIDS, mental illness, and physical disability stigma, and exploring whether and how intersectionality frameworks have been used in the systematic reviews of stigma.

**Methods:**

A search of the literature was conducted to identify systematic reviews which investigated stigma for HIV/AIDS, mental illness and/or physical disability. The electronic databases MEDLINE, CINAHL, EMBASE, COCHRANE, and PsycINFO were searched for reviews published between 2005 and 2017. Data were extracted from eligible reviews on: type of systematic review and number of primary studies included in the review, study design study population(s), type(s) of stigma addressed, and destigmatizing interventions used. A keyword search was also done using the terms “intersectionality”, “intersectional”, and “intersection”; related definitions and descriptions were extracted. Matrices were used to compare the characteristics of reviews and their application of intersectional approaches across the three health conditions.

**Results:**

Ninety-eight reviews met the inclusion criteria. The majority (99%) of reviews examined only one of the health conditions. Just three reviews focused on physical disability. Most reviews (94%) reported a predominance of behavioural rather than structural interventions targeting stigma in the primary studies. Only 17% of reviews used the concept and/or approach of intersectionality; all but one of these reviews examined HIV/AIDS.

**Conclusions:**

The lack of systematic reviews comparing stigma across mental illness, HIV/AIDS, and physical disability indicates the need for more cross-comparative analyses among these conditions. The integration of intersectional approaches would deepen interrogations of co-occurring social identities and stigma.

## Background

Stigma is a dynamic process enacted through structures and individuals, and mediated by relationships of power, control, and domination that are continuously produced and reproduced by actors [1]. At its foundation, stigma is about social inequality and social control, which create a hierarchy that devalues stigmatized people [1].

Stigma is especially problematic for people living with HIV/AIDS (Human Immunodeficiency Virus Infection and Acquired Immune Deficiency Syndrome), mental illness, and physical disabilities because it can create barriers to accessing health care, education, employment, and affordable housing, which in turn, may exacerbate the experience of marginalization [2, 3]. Furthermore, people often live with more than one of these health conditions and may simultaneously experience different kinds of health-related stigma. For example, research indicates that people living with HIV/AIDS have higher rates of depression and anxiety in comparison to the general population [4], and people with physical disabilities are at an elevated risk for depressive symptoms and major depressive disorder [5].

The overlap of different kinds of disease stigma and the rooted-ness of stigma in larger systems of inequality and webs of power have pushed researchers to consider different ways to investigate and analyze it. Given stigma’s links to historical and contemporary manifestations of inequality, power, and systems of domination; intersectionality offers a promising theoretical approach to examine research on stigma. Black feminists, who coined and produced theory on the concept of intersectionality, highlighted how multiple oppression and structural inequalities exist in matrices of domination, which in turn, reinforce unequal relationships of power amongst people; and between people and social institutions such as healthcare, housing, and the law [6–8]. Originally used in feminist theory to describe Black women’s positions within webs of power, intersectionality has been taken up by health sciences researchers to help deepen their analyses of structural and systemic issues in health, and the inequalities and inequities they create [9, 10].

This review of reviews seeks to contribute to the knowledge on stigma by advancing a cross-analysis of HIV/AIDS, mental illness, and physical disability stigma, and exploring whether and how intersectionality frameworks have been used in the systematic reviews of stigma.

## Methods

We adapted Arskey and O’Malley’s [11] scoping review framework to guide the methodology of our review. We used the same subheadings as the authors for the “identifying the research questions” [11] and “identifying relevant studies” [11] stages. However, ‘relevance review’ was used instead of “study selection” [11], and we collapsed “charting the data” and “collating, summarizing and reporting the results” [11] into a single subheading called ‘data extraction, collation and analysis’ to reflect our methodology.

### Identifying the research question(s)

The research questions guiding our review were:

1. What are the characteristics of systematic reviews examining sources of and influences on stigma among those living with HIV/AIDS, mental illness, and/or physical disability?
2. Has intersectionality been used and how has it been used in systematic reviews of stigma and stigma reduction interventions for those living with HIV/AIDS, mental illness, and/or physical disability?

### Identifying relevant studies

The search strategy and electronic database searches were developed and conducted with the assistance of a librarian at the University of Ottawa Health Sciences Library. Electronic databases that publish health-related research and information were accessed. Five databases were searched: MEDLINE, Cumulative Index to Nursing and Allied Health Literature (CINAHL), EMBASE, COCHRANE (Database of Systematic Reviews, EBM Reviews- ACP Journal Club, and EBM Reviews- Database of Abstracts of Reviews of Effects), and PsycINFO.

Several databases indicated that prior to the year 2005, stigma was not a mesh heading, and terms such as “discrimination”, “stereotyping”, and “prejudice” were commonly used. Although we were searching for publications from 2005 and later, we used both the newer and older search terms to ensure that we captured all relevant reviews. The search headings used in all five electronic databases included: “stigma”, OR “prejudice”, OR “social discrimination”, OR “social stigma”, OR “stereotyping”, OR “stereotyped attitudes”, OR “shame”. Keywords used in the search of the databases also included truncated versions of the following terms: discrimination stigma, and prejudice.

Search filters (hedges) were used in the MEDLINE, EMBASE, and PsycINFO databases to limit retrievals to systematic reviews. These filters were not necessary for the COCHRANE database because it only publishes systematic reviews. A filter to restrict retrieved papers to systematic reviews was also applied to the CINAHL database search.

Inclusion criteria for the database searches were:

1. Reviews written in English language
2. Reviews published between January 2005 and November 2017 (inclusive).

Exclusion criteria for the database searches were:

1. Dissertations.

Zotero, a software reference package, was used to manage the citations*.*

### Relevance review

Following the first database search, both authors independently reviewed a sample (*n* = 15) of retrieved titles and abstracts for relevance. They then met to discuss discrepancies in their assessments, and refine the final inclusion criteria for reviews, which were:

- Systematic reviews using qualitative, quantitative, or mixed methods;
- Focus on health-related stigma experienced by study populations with HIV/AIDS, mental illness, and/or physical disability. The definition of mental illness used in the review is consistent with the following definition: “a spectrum of cognitions, emotions and behaviours that interfere with interpersonal relationships as well as functions required for work, at home and in school” [2]. The definition of physical disability used in the review is consistent with the following definition: “any infirmity, malformation or disfigurement that is caused by bodily injury, birth defect or illness and, without limiting the generality of the foregoing, includes diabetes mellitus, epilepsy, a brain injury, any degree of paralysis, amputation, lack of physical co-ordination, blindness or visual impediment, deafness or hearing impediment, muteness or speech impediment, or physical reliance on a guide dog or other animal or on a wheelchair or other remedial appliance or device” [12]
- Stigma is an outcome, result and/or theme of the review and discussed in the research findings; and,
- Review includes measurement tools; conceptual frameworks and theoretical frameworks such as, but not limited to intersectionality; guidance documents; and/or methodological approaches for exploring stigma.

The titles and abstracts of all citations were then screened for relevance by the authors. When relevance could not be ascertained, the full paper was retrieved and reviewed to make a relevance decision.

### Data extraction, collation and analysis

Data were extracted from the reviews using the following categories: aim/objective, specific health issue addressed (i.e. type of mental illness or disability), type of systematic review and number of primary studies included in the review, their geographic location, study design (qualitative, quantitative or mixed methods), study population, type(s) of stigma addressed (interpersonal stigma, intrapersonal stigma, and structural/institutional stigma), and destigmatizing interventions used. We also extracted key findings and recommendations from each review. Data were entered into a table in Microsoft Excel. To ensure that we captured all descriptors of intersectionality, we then did a keyword search of each eligible review paper using the terms “intersectionality”, “intersectional”, and “intersection”. We extracted all definitions and descriptions of these terms from these papers as well as any related findings. We used matrices to compare the characteristics of reviews and their application of intersectional approaches across the three health conditions.

## Results

**Fig. 1:**
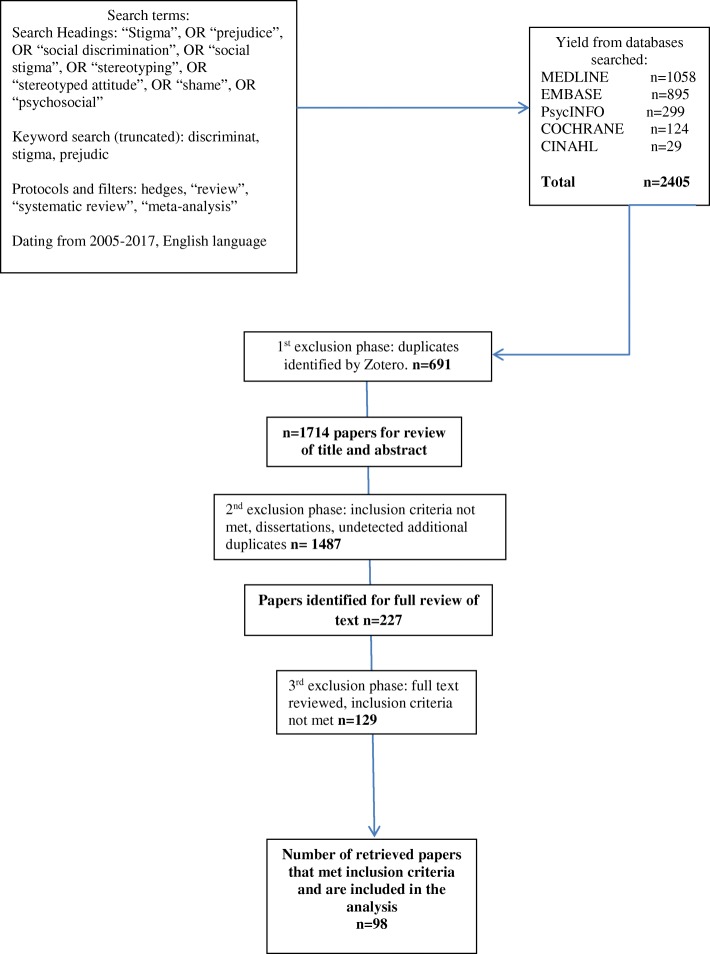
Search results

The electronic database search yielded 2405 citations. In the first exclusion phase, 691 duplicates were eliminated leaving 1714 citations for relevance review. In the second exclusion phase, 1487 papers were eliminated because they were ineligible or found to be additional duplicates**.** In total, 227 papers were identified for a full text review. One hundred twenty-nine papers were found to be ineligible during the third exclusion phase. In total, 98 retrieved systematic reviews of stigma were included in our review (See Fig. 1 for an overview of the search results, and Table 1 for an overview of the reviews).

**Table 1:** Overview of 98 systematic reviews on stigma and HIV/AIDS, mental illness, and physical disability

Author(s), publication year	Title of paper	Type(s) of systematic review	Number of primary studies included in the review	Geographic location(s) of the primary studies
Mental illness				
Abiri et al. (2016) [32]	Stigma related avoidance in people living with Severe Mental Illness (SMI): Findings of an integrative review	Integrative review	21	Africa, Asia, Middle East, Western Europe
Ali et al. (2017) [33]	Perceived barriers and facilitators towards help-seeking for eating disorders: A systematic review	integrative review	13	USA, Australia, Norway, UK, Germany
Ando et al. (2013) [34]	Review of mental-health-related stigma in Japan	integrative Review	19	Japan
Ando et al. (2011) [35]	The simulation of hallucinations to reduce the stigma of schizophrenia: A systematic review	meta-ethnography	11	USA, Australia, Canada
Angermeyer et al. (2011) [36]	Biogenetic explanations and public acceptance of mental illness: Systematic review of population studies	quantitative systematic review with no assessment of methodological quality	33	North America, Asia, South America, Africa, Australia
Boyd et al. (2014) [37]	Internalized Stigma of Mental Illness (ISMI) scale: A multinational review.	quantitative systematic review with no assessment of methodological quality	81	USA
Brohan et al. (2010) [38]	Experiences of mental illness stigma, prejudice and discrimination: A review of measures	quantitative systematic review with assessment of methodological quality	57	Not available
Castaldelli-Maia et al. (2011) [39]	Perceptions of and attitudes toward antidepressant	quantitative systematic review with no assessment of methodological quality	32	USA, UK
Clarke et al. (2014) [40]	Emergency department staff attitudes towards mental health consumers: A literature review and thematic content analysis	integrative review	42	USA, UK, Canada, Australia, Ireland, Sweden, Finland, China, New Zealand
Clement et al. (2015) [41]	What is the impact of mental health-related stigma on help-seeking? A systematic review of quantitative and qualitative studies	integrative review	144	USA, Canada, Europe, Australia and New Zealand, Asia, South America
Clement et al. (2013) [42]	Mass media interventions for reducing mental health-related stigma	COCHRANE quantitative systematic review with assessment of methodological quality	22	Not available
Corrigan et al. (2015) [43]	Do the effects of antistigma programs persist over time? Findings from a meta-analysis	meta-analysis	72	Not available
Corrigan et al. (2012) [44]	Challenging the public stigma of mental illness: A meta-analysis of outcome studies	meta-analysis	72	Europe, North America, South America, Asia, Australia.
Dalky (2012) [45]	Mental illness stigma reduction interventions: Review of intervention trials	quantitative systematic review with assessment of methodological quality	14	Not available
de Mendonca Lima & Lopes (2012) [46]	Systematic review on origin of stigma and discrimination against old persons with mental disorders	integrative review	59	Not available
Doley et al. (2017) [47]	Interventions to reduce the stigma of eating disorders: A systematic review and meta-analysis	quantitative systematic review with no assessment of methodological quality and meta-analysis	18	Australia, US, UK, Indonesia
Edwards et al. (2015) [48]	What do we know about the risks for young people moving into, through and out of inpatient mental health care? Findings from an evidence synthesis	integrative review	40	USA, UK, Finland, Canada, Norway
Ellison et al. (2013) [49]	Bipolar disorder and stigma: A systematic review of the literature	integrative review	25	Public Stigma: UK, Germany, USA, Hong Kong, Malaysia, Canada, Japan, Singapore, Pakistan. Internalized/Affiliative stigma: USA, Canada, Australia, Turkey, UK, South America
Evans-Lacko et al. (2014) [50]	The state of the art in European research on reducing social exclusion and stigma related to mental health: A systematic mapping of the literature	integrative review	97	UK, Finland, Sweden, Germany
Firmin et al. (2016) [51]	Stigma resistance is positively associated with psychiatric and psychosocial outcomes: A meta-analysis	meta-analysis	48	Not available
Gerlinger et al. (2013) [52]	Personal stigma in schizophrenia spectrum disorders: a systematic review of prevalence rates, correlates, impact and interventions	quantitative systematic review with no assessment of methodological quality	54	USA, Europe, North America, Australia, Asia
Griffiths et al. (2014) [53]	Effectiveness of programs for reducing the stigma associated with mental disorders. A meta-analysis of randomized controlled trials	meta-analysis	34	USA, Australia, Hong Kong, Finland, Russia, Turkey
Guruge et al. (2017) [18]	Knowing so much, yet knowing so little: A scoping review of interventions that address the stigma of mental illness in the Canadian context	integrative review	36	Canada
Hanisch et al. (2016) [54]	The effectiveness of interventions targeting the stigma of mental illness at the workplace: A systematic review	quantitative systematic review with assessment of methodological quality	16	Europe, US or Canada, Australia, Asia
Haugen et al. (2017) [55]	Mental health stigma and barriers to mental health care for first responders: A systematic review and meta-analysis	quantitative systematic review with assessment of methodological quality and meta-analysis	12	United States, Ireland, Canada
Hawke et al. (2013) [56]	Stigma and bipolar disorder: A review of the literature	integrative review	32	Not available
Janouskova et al. (2017) [57]	Can video interventions be used to effectively destigmatize mental illness among young people? A systematic review	quantitative systematic review with assessment of methodological quality	23	US, Europe, China, Australia
Jorm (2012) [58]	Belief in the dangerousness of people with mental disorders: A review	integrative review	125	Japan, Germany, Australia, Canada, Turkey, Spain, Nigeria, India, Brazil, Singapore, USA
Kaushik et al.& Kyriakopoulos (2016) [59]	The stigma of mental illness in children and adolescents: A systematic review	integrative review	42	USA, United Kingdom, Ireland, Israel, Australia, Iran, Canada, Greece, Japan.
Kvaale et al. (2013) [60]	Biogenetic explanations and stigma: A meta-analytic review of associations among laypeople	meta-analysis	25	Not available
Kvaale et al. (2013) [61]	The ‘side effects’ of medicalization: A meta-analytic review of how biogenetic explanations affect stigma	meta-analysis	28	Not available
Livingston & Boyd. (2010) [30]	Correlates and consequences of internalized stigma for people living with mental illness: A systematic review and meta-analysis	meta-analysis	127	Not available
Mak et al. (2007) [62]	Meta-analysis of stigma and mental health	meta-analysis	49	North America, Europe, Australia, Asia
Malachowski & Kirsh (2013) [63]	Workplace antistigma initiatives: A scoping study	scoping review	22	Australia, Canada, UK, USA
Mascayano et al. (2016) [63]	Stigma toward mental illness in Latin America and the Caribbean: A systematic review	integrative review	26	Mexico, Brazil, Argentina, Jamaica, Colombia, Peru, Chile
McPherson & Armstrong (2012) [64]	General practitioner management of depression: a systematic review	qualitative systematic review with assessment of methodological quality	13	UK, Sweden, Canada
Mehta et al. (2015) [65]	Evidence for effective interventions to reduce mental health-related stigma and discrimination in the medium and long term: Systematic review	quantitative systematic review with assessment of methodological quality	80	USA, Canada, Australia, UK, New Zealand, Italy, Japan, Norway, Finland, Greece, Hong Kong, Germany, Turkey, Serbia, China, India
Mestdagh & Hansen (2014) [66]	Stigma in patients with schizophrenia receiving community mental health care: a review of qualitative studies	meta-ethnography	18	UK, USA, Europe, Australia, Turkey, Brazil, Malaysia
Milton & Mullan (2014) [67]	Diagnosis telling in people with psychosis	integrative review	14	Not available
Mittal et al. (2012) [68]	Empirical studies of self-stigma reduction strategies: A critical review of the literature	quantitative systematic review with no assessment of methodological quality	14	USA, Canada, Australia, UK, Finland, China
Mueller et al. (2016) [69]	Communications to children about mental illness and their role in stigma development: An integrative review	integrative review	15	UK, Finland, Ireland, Canada, USA, Australia, New Zealand, Spain
Parcesepe & Cabassa et al. (2013) [70]	Public stigma of mental illness in the United States: A systematic literature review	quantitative systematic review with no assessment of methodological quality	36	USA
Putman (2008) [71]	Mental illness: Diagnostic title or derogatory term? (Attitudes towards mental illness) Developing a learning resource for use within a clinical call centre. A systematic literature review on attitudes towards mental illness	integrative review	31	Not available
Read et al. (2006) [72]	Prejudice and schizophrenia: a review of the ‘mental illness is an illness like any other’ approach	quantitative systematic review with no assessment of methodological quality	n/a	USA, England, Australia, Japan, South Africa, Ireland, India, Turkey, Malaysia, China, Italy, Ethiopia, Greece, Russia, and Mongolia
Schnyder et al. (2017) [73]	Association between mental health-related stigma and active help-seeking: Systematic review and meta-analysis	quantitative systematic review with no assessment of methodological quality and meta-analysis	27	Not available
Schomerus et al. (2012) [74]	Evolution of public attitudes about mental illness: A systematic review and meta-analysis	meta-analysis	33	Not available
Seroalo et al. (2014) [2]	A critical synthesis of interventions to reduce stigma attached to mental illness	critical synthesis	17	Hong Kong, USA, Russia, Britain, Germany, Sweden, Turkey
Sharac et al. (2010) [3]	The economic impact of mental health stigma and discrimination: A systematic review	integrative review	30	USA, Germany, Hong Kong, Canada, New Zealand, Israel, UK
Sharp et al. (2015) [75]	Stigma as a barrier to seeking health care among military personnel with mental health problems	quantitative systematic review with assessment of methodological quality	20	USA, UK, Canada
Stubbs (2014) [76]	Reducing mental illness stigma in health care students and professionals: A review of the literature	quantitative systematic review with no assessment of methodological quality	18	Not available
Thornicroft et al. (2016) [77]	Evidence for effective interventions to reduce mental-health-related stigma and discrimination	quantitative systematic review with no assessment of methodological quality	89	Not available
Tsang et al. (2016) [78]	Therapeutic intervention for internalized stigma of severe mental illness: A systematic review and meta-analysis	quantitative systematic review with assessment of methodological quality and meta-analysis	14	US, Canada, Turkey, Hong Kong, Israel, Switzerland, Austria, Netherlands, Japan
Tzouvara & Nyblade. (2016) [79]	Systematic review of the prevalence of mental illness stigma within the Greek culture	quantitative systematic review with no assessment of methodological quality	18	Greece, United Kingdom, Australia, Cyprus
Wittkowski et al. (2014) [80]	Exploring psychosis and bipolar disorder in women: A critical review of the qualitative literature	meta-ethnography	13	USA, Canada, UK, Japan
Wood et al. (2016) [81]	Psychosocial interventions for internalised stigma in people with a schizophrenia-spectrum diagnosis: A systematic narrative synthesis and meta-analysis			
Xu et al. (2017) [82]	Challenging mental health related stigma in China: Systematic review and meta-analysis. I. Interventions among the general public	quantitative systematic review with assessment of methodological quality and meta-analysis	9	China, Hong Kong, Taiwan, Macau
Yamaguchi et al. (2011) [83]	Strategies and future attempts to reduce stigmatization and increase awareness of mental health problems among young people: A narrative review of educational interventions	quantitative systematic review with no assessment of methodological quality	40	Not available
Yamaguchi et al. (2013) [84]	Effects of short-term interventions to reduce mental health–related stigma in university or college students: A systematic review	quantitative systematic review with assessment of methodological quality	35	USA, Taiwan, UK, Japan, Turkey, Germany
Yamaguchi et al. (2017) [85]	Associations between renaming schizophrenia and stigma-related outcomes: A systematic review	quantitative systematic review with assessment of methodological quality	23	Japan, South Korea, UK, China, Canada, Ireland, Turkey
Yang et al. (2014) [86]	Recent advances in cross-cultural measurement in psychiatric epidemiology: Utilizing ‘what matters most’ to identify culture-specific aspects of stigma	integrative review	196	USA, Asia/Pacific Islands, Middle East, Africa, Western Europe
HIV/AIDS				
Bharat (2011) [17]	A systematic review of HIV/AIDS-related stigma and discrimination in India: Current understanding and future needs	integrative review	30	India
Campbell et al. (2011) [87]	Creating social spaces to tackle AIDS-related stigma: Reviewing the role of church groups in sub-Saharan Africa	integrative review	36	Kenya, South Africa, Uganda, Nigeria, Tanzania, Burkina Faso, East Africa, Ghana, Ethiopia, Malawi, Namibia, Zambia, Zimbabwe, Mozambique, Botswana, Senegal, DR Congo,
Columbini et al. (2014) [88]	Factors affecting adherence to short-course ARV prophylaxis for preventing mother-to-child transmission of HIV in sub-Saharan Africa: A review and lessons for future elimination	quantitative systematic review with no assessment of methodological quality	14	South Africa, Ethiopia, Uganda, Zambia, Zimbabwe, Cameroon, Ivory Coast, Rwanda, Kenya
Dao et al. (2013) [89]	Social science research on HIV in Vietnam: A critical review and future directions	qualitative systematic review with no assessment of methodological quality and mixed methods review	64	Vietnam
Darlington & Hutson (2017) [26]	Understanding HIV-related stigma among women in the southern United States: A literature review	integrative review	27	United States
Earnshaw & Chaudoir (2009) [19]	From conceptualizing to measuring HIV stigma: A review of HIV stigma mechanism measures	quantitative systematic review with no assessment of methodological quality	23	USA, South Africa, Kenya, Lesotho, Malawi, Swaziland, Tanzania, Thailand, India, China
Florom-Smith et al. (2012) [90]	Exploring the concept of HIV-related stigma	integrative review	21	USA
Gesesew et al. (2017) [91]	Significant association between perceived HIV related stigma and late presentation for HIV/AIDS care in low and middle-income countries: A systematic review and meta-analysis	quantitative systematic review with assessment of methodological quality and meta-analysis	10	Ethiopia, Venezuala, Mexico, Brazil, Zimbabwe, Kenya
Ho & Holloway. (2015) [92]	The impact of HIV-related stigma on the lives of HIV-positive women: An integrated literature review	integrative review	26	USA, South Africa, Canada, UK, Tanzania, Peru, Vietnam, Kenya, New Zealand, Thailand, India
Katz et al. (2013) [93]	Impact of HIV-related stigma on treatment adherence: Systematic review and meta-synthesis	integrative review	75	Not available
Kerrigan et al. (2015) [16]	A community empowerment approach to the HIV response among sex workers: Effectiveness, challenges, and considerations for implementation and scale-up	meta-analysis.	22	India, Brazil, Dominican Republic
Logie & Gadalla (2009) [94]	Meta-analysis of health and demographic correlates of stigma towards people living with HIV	meta-analysis	24	North America
Lorenc et al. 2011 [95]	HIV testing among men who have sex with men (MSM): systematic review of qualitative evidence	qualitative systematic review with assessment of methodological quality	17	USA, UK, Canada
Loutfy et al. (2015) [21]	Systematic review of stigma reducing interventions for African/Black diasporic women	quantitative systematic review with assessment of methodological quality	5	USA
Lowther et al. (2014) [4]	Experience of persistent psychological symptoms and perceived stigma among people with HIV on antiretroviral therapy (ART): A systematic review	quantitative systematic review with assessment of methodological quality	66	USA, France, Italy, Australia, Canada, Spain, Netherlands, Portugal and Sweden, South Africa, India, Nigeria, Botswana, Brazil, Thailand, Uganda, Cameroon, China, Gambia, Jamaica, Rwanda, Senegal, Vietnam, Zambia
Mahajan et al. (2008) [96]	Stigma in the HIV/AIDS epidemic: a review of the literature and recommendations for the way forward	integrative review	390	North America, Western Europe
Mak et al. (2017) [97]	Meta-analysis and systematic review of studies on the effectiveness of HIV stigma reduction programs	qualitative systematic review with assessment of methodological quality and meta-analysis	m- 42. q s- 35	USA, Africa, Europe, Asia
Maulsby et al. (2014) [27]	HIV among Black Men who have Sex with Men (MSM) in the United States: A review of the literature	integrative review	39	USA
McAteer et al. (2017) [98]	A systematic review of measures of HIV/AIDS stigma in paediatric HIV-infected and HIV-affected populations	qualitative systematic review with assessment of methodological quality	22	United States, Africa, Asia, Sweden
Mills et al. (2006) [99]	Barriers to participation in HIV drug trials: A systematic review	integrative review	14	USA, EU, Australia, France, UK
Monjok et al. (2009) [100]	HIV/AIDS - related stigma and discrimination in Nigeria: Review of research Studies and future directions for prevention	integrative review	8	Nigeria
Monteiro et al. (2013) [22]	The interaction between axes of inequality in studies on discrimination, stigma and HIV/AIDS: Contributions to the recent international literature	integrative review	42	Jamaica, USA, Nepal, Uganda, India, Puerto Rico, England, Mexico, Guatemala, Trinidad and Tobago, Bangladesh, Kenya, South Korea, Dominican Republic, Malawi, China, Tanzania, South Africa, Canada
Monteiro et al. (2012) [101]	Discrimination, stigma, and AIDS: A review of academic literature produced in Brazil (2005–2010)	meta-ethnography and mixed methods review	163	Brazil
Paudel & Baral (2015) [102]	Women living with HIV/AIDS (WLHA), battling stigma, discrimination and denial and the role of support groups as a coping strategy: a review of literature	meta-ethnography	7	Canada, India, Uganda, Australia, Tanzania, United States of America, Thailand
Prost et al. (2008) [103]	Social, behavioural, and intervention research among people of sub-Saharan African origin living with HIV in the UK and Europe: Literature review and recommendations for intervention	integrative review	138	Europe
Roger et al. (2013) [104]	Social aspects of HIV/AIDS and aging: A thematic review	integrative review	62	Canada
Sandelowski et al. (2009) [23]	Gender, race/ethnicity, and social class in research reports on stigma in HIV-positive women	meta-study	32	USA
Sengupta et al. (2011) [105]	HIV interventions to reduce HIV/AIDS stigma: A systematic review	quantitative systematic review with assessment of methodological quality	19	North America, Europe, Asia, Africa
Smith et al. (2008) [106]	A meta-analysis of disclosure of one’s HIV-positive status, stigma and social support	meta-analysis	21	USA, UK, South Africa, India.
Stangl et al. (2013) [15]	A systematic review of interventions to reduce HIV-related stigma and discrimination from 2002 to 2013: How far have we come?	integrative review	48	Saudi Arabia, South Africa, Zambia, China, India, Uganda, Chile, Ethiopia, Australia, China, Ghana, Nigeria, Malawi, Hong Kong, Angola, Cameroon, Ivory Coast, Equatorial Guinea, Kenya, Haiti, Peru, Thailand, USA, Swaziland, Tanzania, Vietnam Canada
Sweeney & Vanable (2016) [107]	The association of HIV-related stigma to HIV medication adherence: A systematic review and synthesis of the literature	quantitative systematic review with no assessment of methodological quality	38	USA, South Africa, Kenya, India, Thailand, China, Tanzania, Hong Kong, Democratic Republic of Congo, Ethiopia, Zambia, Nigeria, France, The Netherlands
Talley & Bettencourt (2010) [108]	A relationship-oriented model of HIV-related stigma derived from a review of the HIV-affected couples literature	integrative review	13	Thailand, India, USA
Turan & Nyblade (2013) [79]	HIV-related stigma as a barrier to achievement of global PMTCT and maternal health goals: A review of the evidence	integrative review	150	Not available
Weihs & Meyer-Weitz (2016) [109]	Barriers to workplace HIV testing in South Africa: A systematic review of the literature	Integrative review	4	South Africa
Physical disability				
Boyles et al. (2008) [14]	Representations of disability in nursing and healthcare literature: an integrative review	integrative review	65	Not available
Wilson et al. (2013) [110]	Attitudes towards individuals with disabilities as measured by the Implicit Association Test: A literature review	quantitative systematic review with assessment of methodological quality	17	USA, China, Italy, UK, Germany, France
Zeldenryk et al. (2011) [111]	The emerging story of disability associated with lymphatic filariasis: A critical review	qualitative systematic review with assessment of methodological quality	16	Ghana, India, Haiti, Sri Lanka, Dominican Republic
Combination papers				
Van Bakel (2007) [13]	Measuring health-related stigma- a literature review	quantitative systematic review with no assessment of methodological quality	63	Most instruments developed for use in the USA

### Characteristics of the systematic reviews

#### Types of systematic reviews included in the review

In total, eight types of reviews were found (Table 2). The most frequent types were integrative reviews (38%, *n* = 37), followed by quantitative systematic reviews with no assessment of methodological quality (17%, *n* = 17), meta-analysis (20%, *n* = 20), and quantitative systematic reviews with an assessment of methodological quality (19%, *n* = 19). There were fewer than five reviews for each of the following categories: meta-ethnography (4%, *n* = 4), qualitative systematic reviews with an assessment of methodological quality (4%, *n* = 4), qualitative systematic reviews with no assessment of methodological quality (1%, *n* = 1), critical synthesis (1%, *n* = 1), scoping review (1%, *n* = 1), meta-study (1%, *n* = 1), and meta-ethnography combined with mixed methods review (1%, *n* = 1).

**Table 2:** Types of systematic reviews, definitions, and references

Type of systematic review	Definition and reference(s)
Critical synthesis	A review which aims to demonstrate that the writer has extensively researched the literature and critically evaluated its quality. This review technique incorporates analysis and conceptual innovation [112].
Integrative review	A technique that integrates review, critique, and synthesis of representative literature on a topic to create new frameworks and perspectives on the topic [113]. It also includes experimental and non-experimental research studies and combines theoretical and empirical data to gain a more comprehensive understandings of a phenomenon [114] .
Meta-analysis	A review technique that “systematically combines the results of quantitative studies to provide a more precise effect of the results” [112].
Meta-ethnography	An interpretive and inductive approach that combines and sometimes compare the findings of ethnographic research or qualitative research to provide a higher level of analysis, generate new research questions, and reduce duplicate research [115, 116].
Meta-study	A research approach that involves the analysis of theory, methods, and findings of qualitative research and synthesizes these insights into new ways of thinking about some phenomena [117].
Mixed methods review	A review technique that combines qualitative and quantitative approaches [112].
Scoping review	A research approach that provides a “preliminary assessment of the potential size and scope” of the research on a particular subject. The aim of this review is to identify the nature and size of research [112].
Qualitative or quantitative systematic review	Engages in a systematized search, appraisal, and synthesis of research evidence which adheres to a set of guidelines [112]. Some qualitative and quantitative reviews include an assessment of methodological quality, while others may not.

#### Disease/condition focus, publication date, and geographic location in the primary studies

Primary studies were reported from over 60 countries; all continents were represented except Antarctica. The majority of the reviews were disease specific, with the largest proportion found for mental illnesses (61%, *n* = 60), followed by HIV/AIDS (34%, *n* = 34); a smaller number of reviews were found for physical disability stigma (3%, *n* = 3). A single review (1%, *n* = 1) looked at stigma across all three health conditions [13] and included other stigmatized health conditions including leprosy, tuberculosis, and epilepsy.

Most reviews of HIV/AIDS and mental illness stigma had been published within the last 5 years (64%, *n* = 63). With only three reviews for physical disability stigma, no publication pattern was discernable.

#### Sample characteristics of the systematic reviews

Across the reviews, the study populations were mostly comprised of people living with one of the three health conditions. For example, people living with HIV/AIDS (PLWHA) were most commonly included in primary research studies on HIV/AIDS stigma (37%, *n* = 22). In the reviews on mental illness stigma, the predominant study populations in the primary studies were people living with mental illness and mental healthcare consumers/users (50%, *n* = 17). There were three reviews in the physical disability stigma category: one examined people living with a physical disability (33%, *n* = 1), the second focused on those who interacted with people living with disabilities (33%, *n* = 1), and the third explored how disability has been considered in nursing and healthcare literature (33%, *n* = 1) [14].

#### Stigma type and interventions included in the primary research

Table 3 shows the number and percentages of each type of stigma investigated in the systematic reviews. Nearly half (47%) of the reviews discussed more than one stigma type, even when the type of stigma described was not an eligibility criterion. Across the health conditions, various stigma types were examined: 78.5% examined intrapersonal forms of stigma (i.e. self-stigma, internalized stigma, perceived stigma, affiliate stigma), 48% of the reviews looked at interpersonal forms of stigma (i.e. social stigma, public stigma, enacted stigma, cultural stigma, experienced stigma), and just 3% focused on institutional/structural stigma (i.e. treatment stigma).

**Table 3:** Types and definitions of stigma discussed in 98 systematic reviews

Stigma Type	Definition	Number	%^a^
Intrapersonal Stigma	When an individual internalizes publicly held negative beliefs about a health condition, and applies them to her or himself	77	78.5
Interpersonal Stigma	The process in which members of the general public direct stigma towards individuals with a specific health condition	47	48
Institutional/Structural Stigma	Practices initiated at the institutional level that work to disadvantage a stigmatized group or person	3	3

Note: ^a^Percentages do not add up to 100 because some reviews included more than one type of stigma

Interventions to manage, reduce, and prevent stigma were included in 36% (*n* = 35) of the reviews. Among these, interventions for mental illness stigma were the most common (74%, *n* = 26), followed by interventions for HIV/AIDS stigma (23%, *n* = 8), and a smaller number of interventions for physical disability stigma (3%, *n* = 1) (See Table 4 for a complete list of interventions and their characteristics).

**Table 4:** Characteristics of the stigma interventions

Author(s), publication year	Health Condition	Institutional Interventions (i.e. interventions administered by hospitals, healthcare institutions, etc.)	Behavioural Interventions (i.e. social contact, education-based, etc.)	Community-based Interventions (i.e. religious, ethno-racial, etc.)
Ando et al. (2013) [34]	Mental Illness		Educational interventions. Contact-based interventions	
Bharat. (2011) [17]	HIV/AIDS			Community mobilisation and involvement.
Clarke et al. (2014) [40]	Mental Illness		Educational interventions	
Clement et al. (2013) [42]	Mental Illness		Mass media	
Dalky (2012) [45]	Mental Illness		Educational interventions contact-based interventions	
Darlington & Hutson (2017) [26]	HIV/AIDS		Support groups, visual media interventions	
Doley et al. (2017) [47]	Mental Illness		Educational interventions. Contact-based interventions	
Gerlinger et al. (2013) [52]	Mental Illness		Cognitive-behavioral therapy Educational interventions	
Griffiths et al. (2014) [53]	Mental Illness		Educational interventions Contact-based interventions	
Guruge et al. (2017) [18]	Mental Illness		Contact-based interventions Experiential videos/photos Educational interventions	Advocacy based
Hanisch et al. (2016) [54]	Mental Illness		Educational interventions Mental Health First Aid (MHFA) training role play, Trauma Risk Management (TRiM), and Crisis Intervention Training (CIT) in first responders.	
Haugen et al. (2017) [55]	Mental Illness		Video	
Hawke et al. (2013) [56]	Mental Illness		Educational interventions Contact-based interventions	
Jorm (2012) [58]	Mental Illness		Educational interventions Contact-based interventions	
Kerrigan et al. (2015) [16]	HIV/AIDS		Educational interventions	Community-empowerment
Loutfy et al. (2015) [21]	HIV/AIDS		Educational interventions Project ACCEPT (Adolescents Coping, Connecting, Empowering and Protecting Together)	
Mak et al. (2017) [97]	HIV/AIDS		Educational interventions	
Malachowski & Kirsh. (2013) [63]	Mental Illness		Educational interventions	
Mehta et al. (2015) [65]	Mental Illness		Contact-based interventions	
Milton & Mullan (2014) [67]	Mental Illness		Educational interventions (Psychoeducation)	
Mittal et al. (2012) [68]	Mental Illness		Educational interventions (Psychoeducation or psychoeducation combined with cognitive restructuring, Psychoeducation with complex multimodal interventions)	
Mueller et al. (2016) [69]			Educational interventions for children	
Parcesepe et al. (2013) [70]	Mental Illness		Contact-based interventions	
Prost et al. (2008) [103]	HIV/AIDS		Educational interventions Prevention interventions.	
Sengupta et al. (2011) [105]	HIV/AIDS		Educational interventions	
Seroalo et al. (2014) [2]	Mental Illness		Educational interventions using theatrical presentations with actors living with mental illness.	
Stangl et al. (2013) [15]	HIV/AIDS	Universal precaution supplies (first aid kits)	Information and skill building	
Stubbs (2014) [76]	Mental Illness		Direct contact, indirect filmed contact, or educational email. Role play	
Thornicroft et al. (2016) [77]	Mental Illness		Education or information interventions, and variants of social contact interventions	
Tsang et al. (2016) [78]	Mental Illness		Psychoeducation	
Wilson et al. (2013) [110]	Physical Disability		Implicit Association Test	
Wood et al. (2016) [81]	Mental Illness		Psychosocial interventions: psychoeducation, thought challenging, connecting with peers and social skills training	
Xu et al. (2017) [82]	Mental Illness		Education interventions either alone or in combination with consumer contact, including contact in person and via video. Education strategies: lectures, role-plays, videos, and educational materials	
Yamaguchi et al. (2013) [84]	Mental Illness		Social contact or video based social contact	
Yamaguchi et al. (2011) [83]	Mental Illness		Contact-based interventions and education interventions	

If the cell is empty, no descriptions were provided

Behavioural interventions such as psychoeducation, informational approaches, and/or social contact were most commonly reported in the reviews of interventions (94%, *n* = 33). In comparison, only one review of HIV/AIDS stigma described structural interventions. In this latter review, a structural intervention (universal precaution supplies to healthcare workers) was combined with a behavioural intervention focusing on information and skill-building to combat HIV/AIDS stigma [15]. Additionally, only 3 reviews described community-based interventions, which included a community-empowerment approach to respond to HIV/AIDS among sex workers [16], community mobilization and involvement to address HIV/AIDS stigma in India [17], and advocacy-based approaches for mental illness stigma in Canada [18].

### Intersectionality

Our text search yielded 13 reviews (17%) whose authors had used an intersectional lens to analyze primary research studies. The majority of these reviews were found in the work on HIV/AIDS stigma (92%, *n* = 12); just one review of mental illness stigma (8%) used intersectionality. While all 13 of these reviews mentioned intersectionality when describing how a mental illness or HIV/AIDS diagnosis intersected with culture, power and/or other differences to reinforce social conditions for stigmatization [19, 20], only three of these 13 reviews (23%) [21–23] provided a definition for intersectionality. Loutfy et al. [21] and Monteiro et al.’s [22] used Crenshaw’s [6, 7] concept of intersectionality in discussing how health inequities are impacted by categories of difference like HIV status, race, gender, and sexuality. Both reviews used this concept of intersectionality to analyze the primary studies and highlighted its usefulness in improving knowledge about how processes of marginalization overlap and are impacted by HIV/AIDS stigma [21, 22]. Sandelowski’s [23] review used a more recent concept of intersectionality, which characterized it as a research paradigm and methodological intervention [24, 25]. For instance, Sandelowski [23] explained that intersectionality acknowledges intra-category diversity and can help to investigate relationships between and among dynamic categories of difference like gender in health research. However, Sandelowski [23] found that non-intersectional, unitary analytical approaches that isolated overlapping categories of difference like race, gender, and HIV stigma from one another were overwhelmingly used in the primary research studies.

#### Intersectional stigma and interventions

Five of the reviews that included intersectionality used it as a framework to discuss the occurrence of intersectional stigma in the primary research studies. This process was defined in Loutfy et al.’s [21] review as the “multiple, simultaneous and dynamic interchanges among categories of social difference as it interlinks with power and privilege, and systemic oppression and its operation at the micro, mesa, and macro levels” (p. 2). The HIV/AIDS reviews tended to focus on stigma amongst groups that have been socially and historically marginalized such as Black women [21, 26], Black men who have sex with men [27], and sex workers [16]. In these reviews, focusing on intersectional stigma allowed other forms of social inequality experienced by people living with HIV/AIDS like racism, sexism, and homophobia to be included and highlighted the existence of overlapping forms of oppression and marginalization.

Although interventions for stigma were described in 36% of the reviews, none of the authors indicated whether or how intersectional interventions were used in the primary studies. However, three of the 35 reviews (8%) highlighted the lack of intersectional interventions designed to address intersectional stigma in the primary studies, and advocated for their use in primary research studies [15, 21]. Loutfy et al.’s [21] review found there was an absence of stigma-reducing interventions that addressed co-occurring stigmas experienced by Black women who are HIV positive, and that most focused on interpersonal and intrapersonal stigma. Kerrigan et al.’s [16] review of community-empowerment interventions to counter HIV stigma amongst mainly female sex workers found that one of the greatest structural barriers to the implementation and scale-up of these interventions was the presence of intersectional stigma.

## Discussion

To our knowledge, this is the first review to provide a cross-analysis of systematic reviews of HIV/AIDS, mental illness, and physical disability stigma. It is also the first review of reviews to examine whether and how intersectionality has been used as an analytic approach on stigma.

The nearly complete lack of reviews that examined stigma across these three health conditions may be partly attributed to trends in stigma research [28], since systematic review topics are constrained by primary studies on a topic. Researchers conducting effectiveness studies of interventions concentrate on specific target groups and conditions, in part because the aim of effectiveness research is to determine whether a specific outcome can be attributed to a particular intervention. Thus, researchers doing effectiveness studies are more likely to focus on homogenous (e.g. populations with a primary diagnosis such as HIV/AIDS) rather than heterogeneous populations. There is also a predominance of disease-specific funding, which may preclude and/or discourage cross-analytical work. Funding calls that require stigma research on heterogeneous samples would foster this kind of work, enabling cross-analyses of stigma for these health conditions.

We noticed that in reviews of intervention studies, there is a dominant focus on behavioural rather than structural interventions for stigma, and that reviews typically focus on interpersonal and intrapersonal stigma rather than structural and institutional stigma. This gap may be due to a dearth of primary studies with a structural focus, reflecting a persistent person-centric orientation towards stigma reduction [29]. In addition to the need for more reviews that compare stigma and destigmatizing interventions across disease conditions, there is a need for reviews that compare behavioural and structural interventions or their combination. This would provide an important basis for comparing stigma reduction approaches with either (or both) orientations.

Our review indicates that research on stigma has begun to move in the direction of acknowledging the intersectionality of these experiences and grappling with how stigmatization overlaps with other forms of oppression. Although just one of the mental illness stigma reviews used intersectionality, it highlighted the potential value of intersectionality as an analytical framework, noting that it captured the interlocking effects of various kinds of oppression as it overlaps with mental illness stigma [30]. A small proportion of systematic reviews of HIV/AIDS stigma were categorized as using intersectionality frameworks. We think the integration of intersectionality within some HIV/AIDS reviews could be a result of the expansive body of diverse and cross-cultural, cross-racial, and cross-geographical research on HIV/AIDS stigma, which may have stimulated the application of analytical frameworks that acknowledge the convergence of multiple kinds of stigma and structural inequality.

The limited number of reviews addressing physical disability stigma is noteworthy. We do not know if this gap reflects a lack of primary research studies on this topic. Nevertheless, Boyles et al. [14] stated that historically, most research on disability has been designed, conducted, and managed by people who do not live with disabilities, which limits knowledge creation about these health conditions. This is in sharp contrast with research on HIV/AIDS, which has a long history of involving persons living with HIV/AIDS in many facets of research studies. If more opportunities were created to meaningfully include and facilitate leadership roles for people living with disabilities in health research, we might see an increase in both primary research and systematic reviews on this topic. Purposefully engaging those living with physical disability in studies on HIV/AIDS and mental health would help to build the evidence-base on co-occurring stigma.

### Recommendations for future research

This review of reviews indicates there is a need for more work that focuses on structural interventions to reduce stigma in both primary studies and systematic reviews. While several reviews highlighted the lack of structural interventions in their findings, we believe it is also imperative to identify concrete examples of these kinds of interventions when reviewers present recommendations for future work on this topic. At the organizational level, examples might include research that examines institutional interventions implemented to reduce stigma in healthcare settings like culturally-specific mental healthcare programs, clinical assessments that omit problematic or pathologizing questions about gender and sexuality, and anti-stigma training for healthcare professionals. At the state level, policies such as legalizing/decriminalizing homosexuality or enacting legislation that protects the rights of people living with disabilities and mental illness are examples of structural interventions that might be expected to have an impact on stigma.

To strengthen the reviews of stigma across these health conditions and in cross-comparative work we call for more explicit integration of intersectionality frameworks in the methodology of systematic reviews. It is not sufficient for reviews to use the language of intersectionality as an afterthought in the conclusion or discussion sections or to hint at co-occurring inequalities or people’s multiple social identities without context, clear definitions, and critical reflections on how the term has evolved. Researchers need to work towards more accurate and meaningful inclusions of intersectional approaches, which use the concept to deepen analyses of stigma, particularly as it applies to understanding the presence of stigma from more than one health condition and from other co-occurring sources of stigma emanating from social identities like race, gender, and sexuality.

### Limitations

We searched five reputable databases that publish medical and health research. However, we did not search databases that are exclusively in the social sciences and may have missed some pertinent reviews as a result. We did not use intersectionality as a search term. This may have led to the omission of some eligible reviews although that seems unlikely since our broader search terms likely captured any reviews that included intersectional approaches. Nevertheless, the patterns we observed are overwhelming and it seems unlikely that a different pattern would have emerged even if some additional reviews had been found.

We were cautious in our categorization of reviews as using or not using intersectional approaches, and reviews that included a discussion or analysis of other social categories like race or gender were not categorized as intersectional on that basis alone. Our conservative approach is consistent with a literature that warns against misappropriation of the concept, describing how it is sometimes used to gloss over identity politics in research or to treat categories like race and gender as independent variables rather than as reflections of social practices that are linked to larger processes of inequality [9, 30, 31]. We acknowledge that more conventional systematic review methods may not be congruent with reporting on this deeper and more nuanced approach to the intersectional analyses of stigma.

Our approach to identifying and selecting articles focused on reviews rather than primary studies. While it seems reasonable to conclude that the gaps we identified from systematic reviews mirror gaps among primary studies, we are not able to confirm this.

## Conclusions

The nearly total lack of systematic reviews examining stigma across mental illness, HIV/AIDS, and physical disability indicates there are ripe opportunities for further primary research and systematic reviews that undertake a cross-comparative analyses among these health conditions. Approaches such as intersectionality that deepen our interrogation of intersecting stigma and that acknowledge and address larger processes of inequality and inequity that occur alongside health stigma are needed. Such approaches may inform intervention design as well as research methods; these are needed to avoid reproducing and exacerbating inequalities and inequities among population that experience marginalization due to their health condition(s).

## Abbreviations

CINAHL: Cumulative Index to Nursing and Allied Health Literature
HIV/AIDS: Human Immunodeficiency Virus Infection and Acquired Immune Deficiency Syndrome
PLWHA: People living with HIV/AIDS
